# Feasibility of in-home electroencephalographic and actigraphy recordings in dogs

**DOI:** 10.3389/fvets.2023.1240880

**Published:** 2024-01-08

**Authors:** Emily Folkard, Charly McKenna, Gabrielle Monteith, Lee Niel, Luis Gaitero, Fiona May Keir James

**Affiliations:** ^1^Department of Clinical Studies, University of Guelph, Guelph, ON, Canada; ^2^Department of Population Medicine, University of Guelph, Guelph, ON, Canada

**Keywords:** canine epilepsy, idiopathic epilepsy, behavioral comorbidities, questionnaires, electroencephalography, actigraphy

## Abstract

**Introduction:**

Idiopathic epilepsy is a prevalent neurological disease in dogs. Dogs with epilepsy often present with behavioral comorbidities such as aggression, anxiety, and fear. These behaviors are consistent with pre, post, or interictal behaviors, prodromal changes, seizure-precipitating factors, or absence and focal seizures. The overlap in behavior presentations and lack of objective research methods for quantifying and classifying canine behavior makes determining the cause difficult. Behavioral comorbidities in addition to the task of caring for an epileptic animal have a significant negative impact on dog and caregiver quality of life.

**Methods:**

This pilot study aimed to assess the feasibility of a novel technology combination for behavior classification and epileptic seizure detection for a minimum 24-h recording in the dog's home environment. It was expected that combining electroencephalography (EEG), actigraphy, and questionnaires would be feasible in the majority of trials. A convenience sample of 10 community-owned dogs was instrumented with wireless video-EEG and actigraphy for up to 48 h of recording at their caregiver's home. Three questionnaires (maximum 137 questions) were completed over the recording period by caregivers to describe their dog's everyday behavior and habits.

**Results:**

Six of the 10 included dogs had combined EEG and actigraphy recordings for a minimum of 24 h.

**Discussion:**

This shows that in-home EEG and actigraphy recordings are possible in community-owned dogs and provides a basis for a prospective study examining the same technology combination in a larger sample size.

## Introduction

Epilepsy is a common neurological disease that dogs present with in veterinary medicine (1). Idiopathic epilepsy (IE), where a cause for the disease cannot be determined, has an estimated prevalence of 0.6% in the general population of companion dogs (1, 2). As seen in people, dogs with epilepsy often experience behavioral changes throughout the course of the disease, including increased comorbid anxiety, aggression, fear, and clinginess (3, 4). Additional behavioral changes as a result of anti-seizure drug usage, include lethargy, lack of motor control, polyuria, and polydipsia (5, 6). Changes in behavior also occur with ictal events. For example, absence or focal seizures experienced in IE are commonly accompanied by altered awareness, lip smacking, facial twitching, and excessive blinking (6). These may be misinterpreted as abnormal behaviors instead of ictal events, contributing to their underreporting (7). Despite the growing recognition of behavioral changes in dogs with epilepsy and the complexity of behavior designation, limited options exist to aid in the classification of these behaviors.

Various investigative tools have been used to enhance our understanding of the impact of epilepsy on canine behavior. Caregiver-reported questionnaires have been used in a variety of contexts including quality of life concerns, seizure semiology, anti-seizure drug side effects, and behavioral changes at various time points throughout the disease (8–13). Although useful for providing initial insight for future investigations, caregiver-reported questionnaires are subjective in nature and prone to recall and observer bias (14). Thus, objective tools are required to provide an accurate depiction of canine behavior within epilepsy.

Electroencephalography (EEG) is instrumental in the diagnosis and treatment of canine epilepsy, with promise for investigation of behavior changes and potential underlying causes. Historically, EEG on sedated or anesthetized animals prevented concurrent analysis of behavior (15–19). Recent ambulatory successes make it the only method with adequate spatial and temporal resolution for accurately diagnosing and classifying seizure types in awake and behaving dogs but only one report of recording in a dog's home environment (20). When EEG is combined with synchronized video recording (vEEG), brain activity captured by EEG can be correlated to behaviors captured on video to aid in objective behavior classification.

As behavior classification using vEEG is time and labor intensive, it would improve clinical and research efficiency to augment EEG with an automated tool. Actigraphy uses accelerometer technology to measure rest and activity levels, and algorithmic analysis of accelerometer data has been used to identify normal behavioral states in dogs such as walking, sleeping, head shaking, and eating (21). Algorithms generated using accelerometer data have successfully identified generalized tonic-clonic (GTC) seizures in dogs, but have been unable to detect non-GTC seizures (21–23). It is possible, then, that combining the ability of EEG to detect and classify all seizure types with the behavior-classifying ability of accelerometers would aid in our understanding and objective classification of behavior within canine epilepsy. The further addition of caregiver-completed behavioral questionnaires would supplement insight into the dog's everyday behavior to aid in distinguishing normal from abnormal behaviors.

This pilot study assessed whether a novel combination of vEEG, actigraphy, and caregiver-reported behavioral questionnaires can be used to collect a minimum 24-h recording in IE and neurotypical (NT) companion dogs at the caregiver's home. Both IE and NT dogs were included to ensure there were no major differences in feasibility between the groups to allow for behavioral comparisons to be made in prospective studies utilizing the data collected in the current study. We predicted that combining vEEG, actigraphy, and questionnaires for a minimum 24-h at-home recording would be a feasible approach to capturing a complete and detailed account of seizure activity and canine behavior to enhance our understanding of canine behavior in epilepsy.

## Materials and methods

### Subjects

All participants were recruited through the Ontario Veterinary College Health Science Center (OVC HSC) Neurology Service, regional neurology practices, and the research program's social media channels. The study protocol was reviewed and approved by both the Research Ethics Board and the Animal Care Committee at the University of Guelph (REB#22-05-12, AUP#4695). All caregivers provided informed consent.

Inclusion criteria for recruitment were IE and NT dogs between the ages of 2 and 8 years old to help reduce adolescent and geriatric-related behavioral and cognitive differences. IE dogs were required to have been previously diagnosed with minimum tier I IE based on the International Veterinary Epilepsy Task Force (IVETF) recommendations (24). Tier I IE encompasses dogs with (I) a history of two or more unprovoked seizures occurring at least 24 h apart, (II) age at seizure onset between 6 months and 6 years, (III) normal interictal physical and neurological examinations, and (IV) no clinical abnormalities on laboratory tests (including biochemistry profile and complete blood count, with or without fasting bile acids or ammonia, and urinalysis) (24). Aside from tier I IE, both IE and NT participants were required to have an unremarkable medical history with no diagnosed behavioral disorders. Physical and neurological examinations were completed for all dogs by members of the OVC HSC Neurology Service and were required to be unremarkable at the time of study enrolment for inclusion. Dogs were excluded if they were deemed unfit for vEEG instrumentation due to their tolerance for handling.

### Instrumentation

Instrumentation for vEEG and actigraphy was completed at the OVC HSC. Following physical and neurological examinations, dogs were sedated, if required, for electrode placement using our standard clinical protocol; dexmedetomidine 10–20 μg/kg intravenous (IV) for sedation with atipamezole 100–200 μg/kg intramuscular (IM) for subsequent reversal. If the dogs required sedation, the official recording start time was extended beyond clinical recovery until normal behavior and mentation was observed.

**Figure 1:**
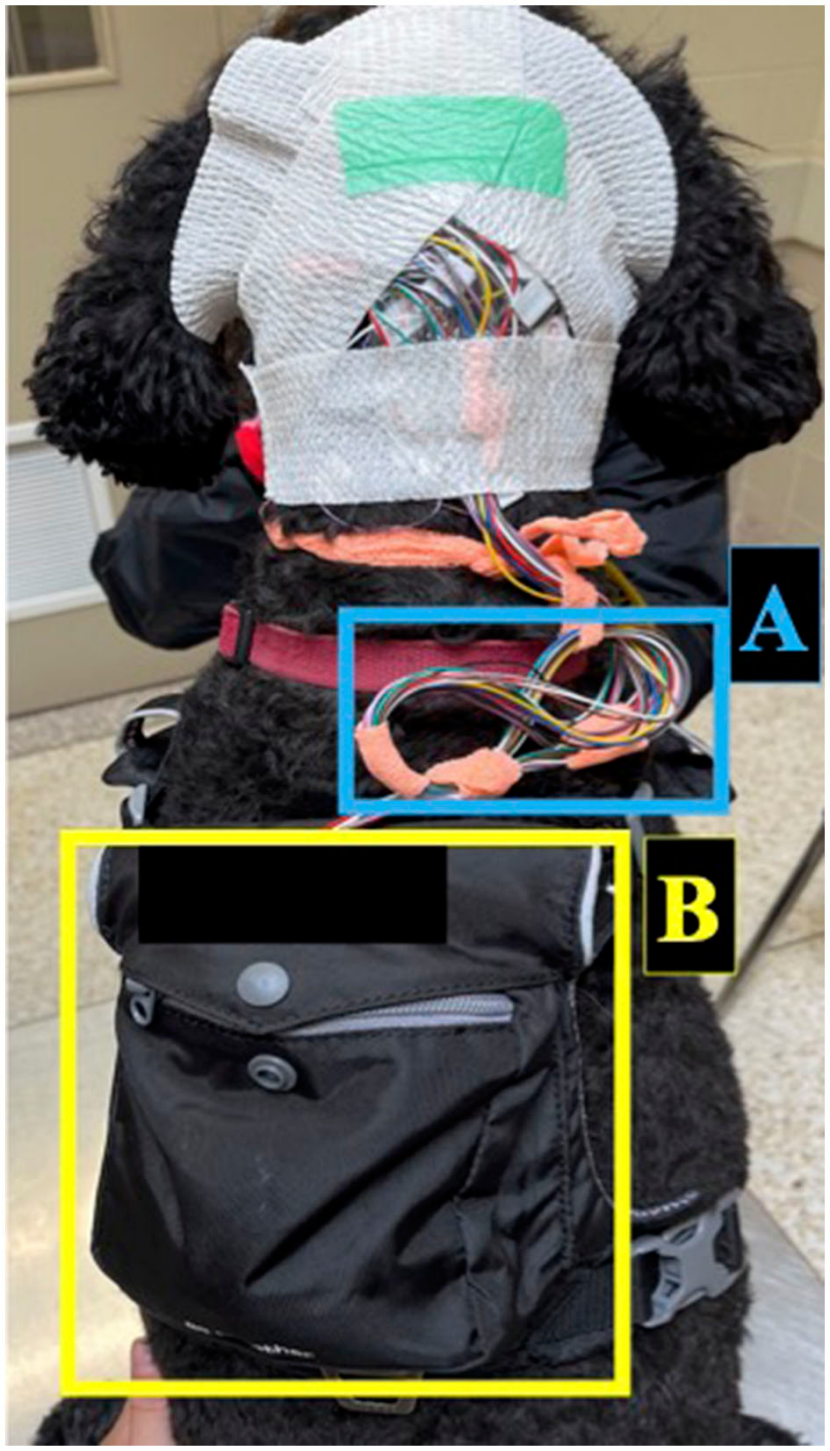
Set-up for ambulatory EEG recording in dogs. Electrodes were secured to the scalp using sticky bandage and connected to leads which were secured with non-adhesive bandage. The leads were managed with a tension loop **(A)** and connected to the ambulatory EEG amplifier secured in the dorsal pocket of a harness **(B)**.

All vEEGs were conducted using 15 subdermal wire electrodes following our previously described electrode placement protocol, with even numbers denoting right side electrodes and odd numbers denoting left side electrodes (Table 1) (25). Electrodes included reference (R), ground (G), midline (Fz, Cz, Pz), frontal electrodes (F3/F4, F7/F8), central electrodes (C3/C4), temporal electrodes (T3, T4), and occipital electrodes (O1/O2). During instrumentation, electrodes were adjusted to keep impedances below 30 kOhms. Electrodes were secured to the scalp using sticky bandage and connected to leads plugged into the Lifelines Neuro Trackit T4A ambulatory EEG amplifier (Lifelines Neuro Company, Louisville, USA) secured in the dorsal pocket of a harness worn by the dog. Non-adhesive bandage was used to secure the leads in place on top of the dog's head, and a tension loop was created with the leads and non-adhesive bandage to reduce tension on the electrodes (Figure 1). Two hours were given to allow the dog to acclimatize to the equipment before being sent home.

**Table 1:** Electrode placement protocol.

**Electrode**	**Electrode location**
R	Midline, between medial canthi
G	Dorsal midline neck, 2–5 cm caudal to occipital protuberance
F7/F8	Zygomatic arch just caudal to the lateral canthus of both eyes
F3/F4/Fz	On the temporal lines causal to the medial canthi and at the midline
C3/C4/Cz	Halway between F and O/P electrodes, in line with T electrodes
O1/O2/Pz	Transverse line between mastoid processes in line with F electrodes
T3/T4	Zygomatic arch, just rostral to the pinnal edge

The vEEG amplifier was connected via Bluetooth to a laptop with synchronous webcam video recording. Following instrumentation, caregivers were sent home with the laptop for up to 48 h of at-home vEEG monitoring. Caregivers were instructed to keep the webcam trained on the dog as much as possible to allow for behavior and/or ictal event confirmation. Caregivers were advised that the EEG and concurrent video recording could be stopped at any time for any reason at their discretion and were able to arrange a time to return for de-instrumentation at their convenience. Otherwise, recordings were terminated when the dog removed the equipment spontaneously or after 48 h.

**Figure 2:**
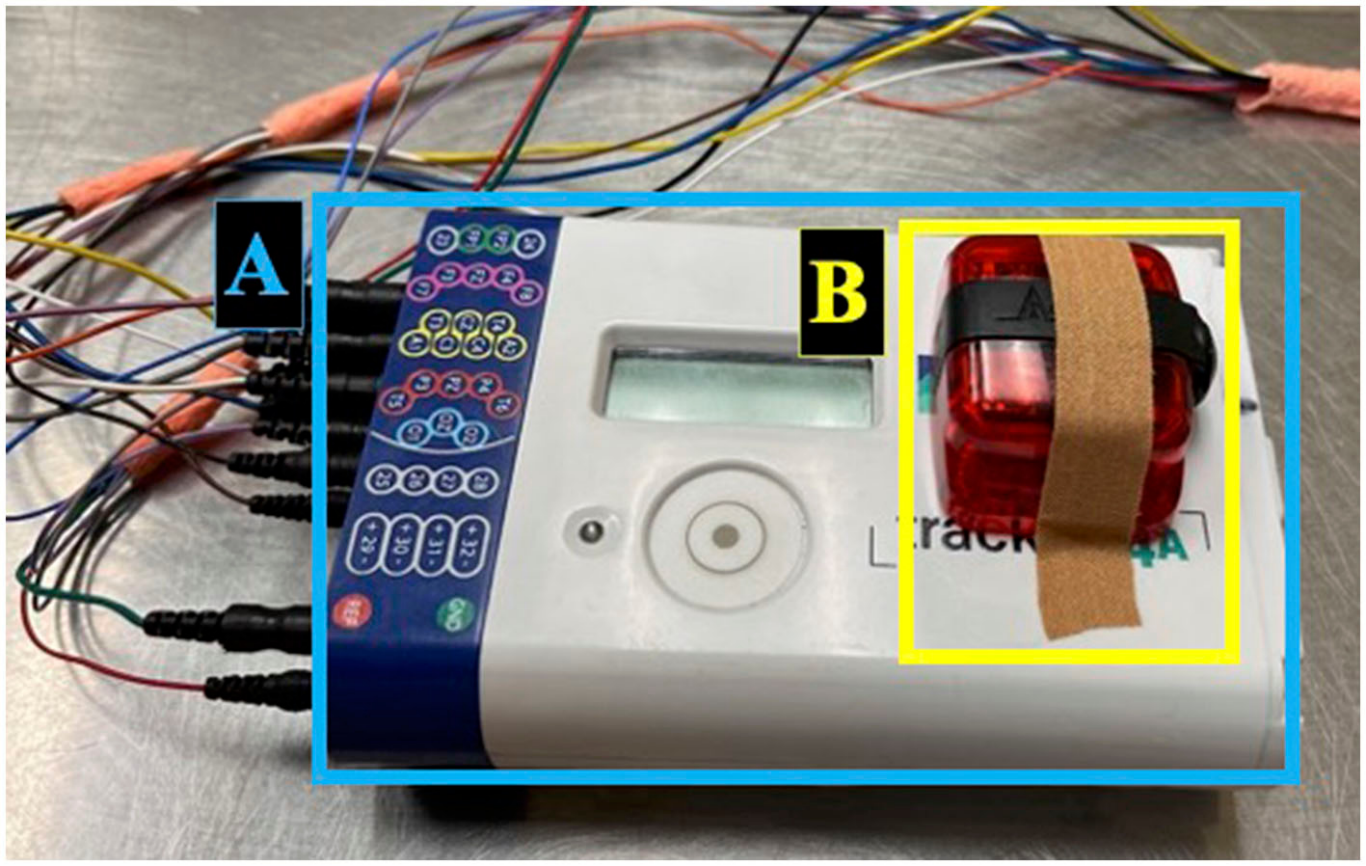
Set-up for ambulatory EEG recording in dogs continued. EEG amplifier **(A)** located inside the dorsal pocket of the harness. Actigraph unit secured on the EEG amplifier using sticky bandage **(B)**.

The Actigraph GT9X Link (Actigraph, Pensacola, USA) was secured to the EEG amplifier using sticky bandage inside the harness pocket located on the dog's interscapular region (Figure 2). The Actigraph GT9X Link was chosen as it was provided through our collaboration for this project and fit within the harness pocket. To prevent the rotation of the actigraph, the harness was chosen based on body size and adjusted accordingly. The actigraphs were initialized to the proximity setting to allow for both monitoring of activity levels and the dog's proximity to key locations in their environment. Three additional actigraph units were dispensed to caregivers for proximity measures; (I) to be worn by the caregiver on their wrist or hip to monitor the dog's proximity to the caregiver, (II) to be placed at the dog's water bowl to estimate water consumption, and (III) to be placed at the exit door the dog used most frequently to access the outdoors to monitor time spent outside. Once placed at these locations, caregivers were requested not to move the actigraphs until the end of the recording. The actigraph recordings were initialized such that recordings would not commence until after the caregiver had returned home and placed the units in their respective locations and were terminated once the caregiver removed the units from these locations or after 48 h.

### Questionnaire design

Using Qualtrics (Qualtrics, Provo, USA), three questionnaires were implemented throughout the study and completed online by the caregiver. The first questionnaire (Q1) (Supplementary material 1) included 137 questions related to the dog's typical behavior and sleep, housing, care, exercise habits, medical history, and seizure signalment (if applicable). Questions pertaining to the dog's behavior and sleep were copied from the previously validated Canine Behavioral Assessment and Research Questionnaire (CBARQ) and Sleep and Night Time Restlessness Evaluation Score (SNoRE), respectively (10, 26). Q1 was completed at the OVC HSC during the acclimatization period.

The second and third questionnaires (Q2 and Q3, respectively; Supplementary material 2) were identical condensed versions of Q1 that were to be completed after every 24 h of recording. If the recording terminated before 24 h or between 24 and 48 h, Q2 or Q2 and Q3 were not completed. Questionnaires 2 and 3 were comprised of 125 questions each.

All 3 questionnaires were completed by outside reviewers prior to enrolment to help ensure the questions were clear and could be completed in a reasonable time (< 30 min). Completion rate and time were collected using features embedded in Qualtrics for further analysis.

### Feasibility criteria

The following feasibility criteria were assessed to determine success or failure for each dog: (I) a >24-h readable EEG and actigraphy recording, (II) a minimum of 2 electrodes remaining in place on each of the right, left, and midline regions, (III) the actigraph remained secure for the duration of the recording, (IV) the dog was able to complete normal tasks with no concerns as reported by the caregiver in questionnaires, and (V) the caregiver was able to complete the questionnaires in reasonable time with no concerns. All feasibility criteria had to be satisfied for a session to be deemed successful.

### Statistical analysis

Raw vEEG data was analyzed using Persyst 12 (Persyst Development Corporation, San Diego, USA) and raw actigraphy data was analyzed using Excel 2016 (Microsoft Corporation, Redmond, USA). Simple descriptive statistics were performed on the feasibility outcome measures including EEG and actigraphy recording duration, electrode survival times, and questionnaire completion rate and duration for Q1, Q2, and Q3. Standard error was reported with means to adjust for sample size. A Friedman's rank test was performed to compare the effect of electrode placement location on electrode survival time. A two-sample *t*-test was used to compare the mean EEG recording duration time between sedation and not sedated groups. A Shapiro Wilk test and examination of the residuals confirmed the data was normally distributed. Precision was calculated for measured parameters to determine the prediction limits for the difference in vEEG recording duration between sedated and non-sedated dogs with a power of 85% was calculated as well as the actual power with four and six dogs. All statistical analyses were conducted in R Statistical Software (v4.2.2, R Core Team 2022) and SAS version 9.4 (SAS Institute Inc, Cary, USA).

## Results

Six IE and 5 NT dogs were recruited for a total of 11 dogs. One NT dog was excluded due to difficulties with handling during the physical and neurological examinations. Therefore, 6 IE dogs and 4 NT dogs were included for a total of 10 dogs. Participating breeds included six mixed breed dogs and one of each of the following: Border Collie, Standard Poodle, Golden Retriever, and Black Mouth Cur (Supplementary material 3). There were five neutered males and five spayed females with a median age of 5 years (range 2–8 years).

Six of 10 sessions (60%) met all feasibility criteria and were therefore considered to be a success. Five trials terminated at or around 48 h as scheduled. Four trials were terminated prior to 48 h due to spontaneous unscheduled de-instrumentation by the dog and one trial was terminated prior to 48 h due to caregiver concerns of growing discomfort for their dog. There were no caregiver concerns regarding the safety of the biomonitor suite for at-home usage and the possibility of wearing it during seizure activity. The following subsections describe each feasibility outcome in depth.

### vEEG

The feasibility of vEEG recordings was determined based on the number of functioning electrodes that remained in place for the duration of the recording. Two electrodes each on the left side, midline, and right side were to be secure and reliably recording for >24 h for an EEG to be considered successful. All vEEG recordings were of sufficient interpretable quality while the required number of electrodes were in place despite frequent muscle and movement artifacts and occasional electrode “pops.” Due to technical issues, a very limited amount of video was captured alongside the EEG recording. This limited video was used to confirm stages of mentation on EEG output but was omitted from further analysis.

Six of 10 dogs required sedation for electrode placement using dexmedetomidine (10–20 ug/kg) and subsequent reversal with atipamezole (100–200 ug/kg). The remaining dogs (4/10) had instrumentation successfully completed without sedation. One sedated dog experienced a clonic seizure during instrumentation and was administered diazepam (0.5 mg/kg IV) as a rescue medicine to stop the episode. Recordings began once clinical recovery and normal behavior and mentation was achieved.

Six out of the 10 EEG recordings were >24 h in duration and were therefore considered successful. The duration of EEG recording in total ranged from 1.90 to 48.27 h, with a median duration of 27.70 h. Of recordings that lasted >24 h, the median recording duration was 46.75 h (range: 27.7–48.27). Recordings that did not reach 24 h had a median recording duration of 2.55 h (range: 1.90–17.42). Recording duration was similar between dogs that were sedated for instrumentation and unsedated dogs, with mean recording durations of 19.20 (standard error: 7.23) and 42.02 h (standard error: 8.85), respectively (*p* = 0.08). Data was normally distributed, confirmed by the Shapiro Wilk test (*p* = 0.113). As the two-sample *t*-test was underpowered with six unsedated dogs and four sedated dogs, we calculated that sufficient power to detect a potential difference in recording duration between sedated and unsedated dogs would be achieved with eight dogs in each group. Reasons for early recording termination were spontaneous removal of the EEG by the dog (*n* = 3) and caregiver preference (*n* = 1). Notably, there was no damage sustained to the EEG transmitter unit, leads, harness, or actigraph unit during this study. Also, neither ictal nor interictal epileptogenic paroxysmal discharges were recorded in the IE group.

**Figure 3:**
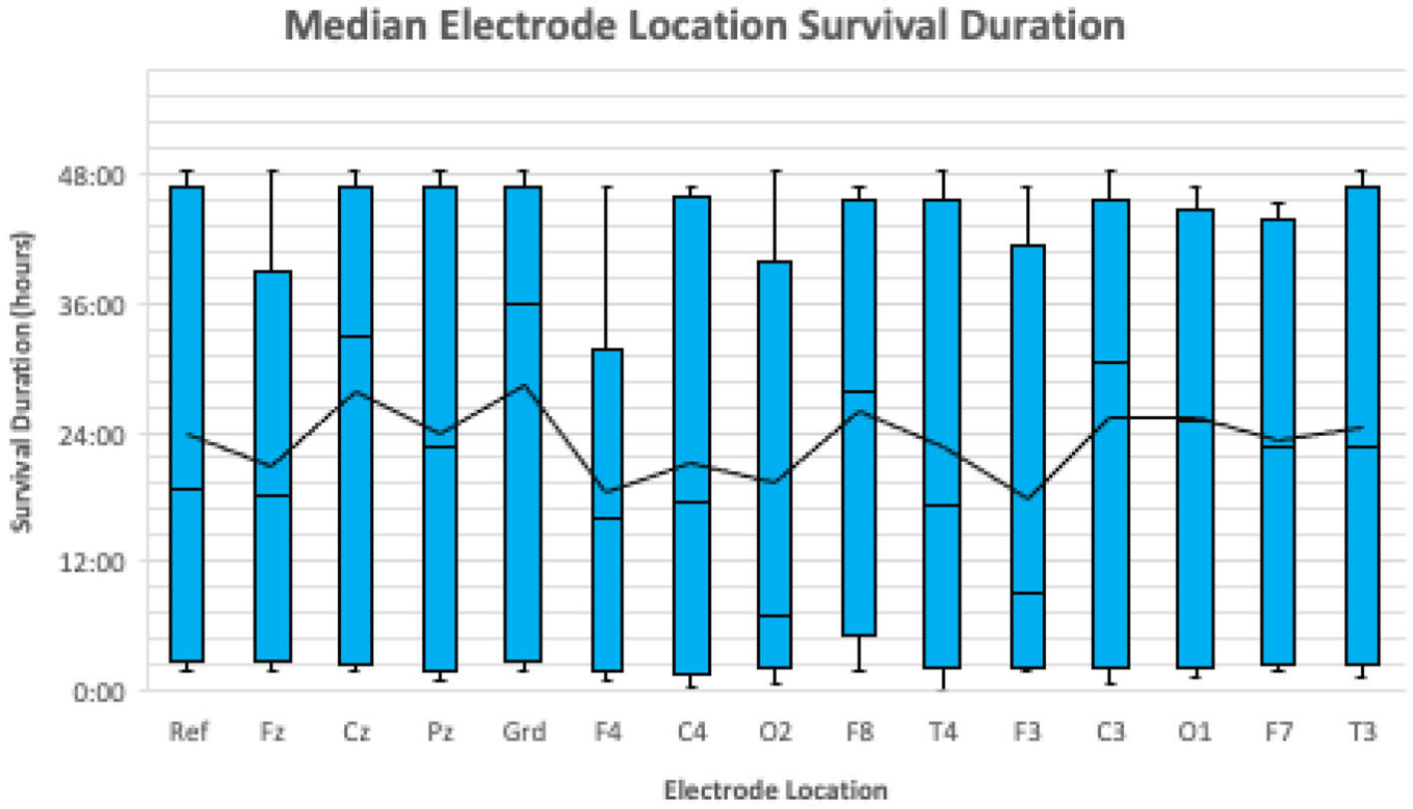
A boxplot illustrating the median survival duration of each electrode location for all 10 dogs. The median survival duration for each location is represented by the horizontal black line in each blue box. The mean survival duration for each location is represented by the continuous black line.

Electrodes were considered lost when the cortical signal captured by the electrode was no longer interpretable due to artifact. The median proportion of electrodes lost by the end of the EEG recording was 40.0% (range: 13.0–64.0%). There was no significant difference in the odds of losing an electrode between sedated and non-sedated dogs [OR = 1.06, 95% CI (0.049–23.16)]. The sample was too small to detect a statistically significant difference in mean electrode survival time between electrode placement locations (Friedman's *p* = 0.733; Figure 3).

### Actigraphy

Actigraphy recordings were considered successful if their duration was >24 h and the unit remained securely in place next to the EEG transmitter unit inside the harness pocket on the dog's back.

Six out of 10 actigraphy recordings were >24 h in duration and were therefore considered successful. Actigraphy recording durations ranged from 1.72 to 48.00 h, with a median recording duration of 25.62 h. Of actigraphy recordings that lasted >24 h, the median recording duration was 46.13 h (range: 21.53–48.00) and the recordings that did not last 24 h had a median duration of 5.27 h (range: 1.72–8.92). Actigraphy units remained secure in their original location attached to the EEG transmitter unit on the dog's back in all 10 dogs. Actigraphy recordings in all 10 dogs were terminated near the end of EEG recordings when caregivers moved the units from their homes prior to de-instrumentation.

### Questionnaires

All 10 caregivers completed Q1 at the OVC HSC during the acclimatization period with a completion rate of 100%. Completion times ranged from 21.20 to 152.30 min, with a median completion time of 101.47 min.

Q2 and Q3 were completed at home by caregivers after 24 and 48 h of the recording. Caregivers were instructed not to complete the questionnaires if the recording had been terminated before the 24- or 48-h mark. Q2 was completed by 9/10 participants with a completion rate of 100%. Completion times ranged from 12.40 to 2,748.93 min, with a median completion time of 16.5 min. Q3 was completed by 6/10 participants with a completion rate of 100%. The median completion time for Q3 was 21.94 min and ranged from 8.72 to 383.02 min.

## Discussion

Three tools commonly used in the investigation of canine epilepsy and behavior are EEG, actigraphy, and caregiver-completed questionnaires. This study demonstrated for the first time that EEG can be successfully recorded in the home environment of companion dogs, although the synchronized video technology proved unreliable. In a further first, this study combined EEG home-recording of dogs with actigraphy for a minimum of 24-h recording in addition to the completion of caregiver-reported behavioral questionnaires. This combination of technologies appeared feasible as median EEG and actigraphy recording times of 27.70 and 25.62 h, respectively, were achieved in 60% of participants. All questionnaires that were attempted by caregivers were completed to 100%, indicating that the surveys were not too onerous for motivated participants.

There was a bimodal “survival time” for the wearable study biomonitors. There was a difference of ~44 h median recording duration between EEG recordings lasting >24 h and those lasting less. Similarly, the difference in median actigraphy recordings lasting >24 h and those lasting less was ~37 h. These large differences may be due to canine patient-specific factors such as age, weight, medication status, and/or behavioral characteristics. The influence of these various factors provides the groundwork for a prospective study investigating the correlation between EEG and actigraphy recording length and the dog's behavioral profile, given that four dogs spontaneously de-instrumented themselves. It is noted that the dog's behavior may be impacted by the bulkiness of the equipment thus this factor should be considered in future investigations of behavior. Furthermore, understanding canine patient-specific factors that influence EEG and actigraphy recording length would help determine if these biomonitor technologies are suitable diagnostic options for each patient to optimize clinical efficiency and support personalized patient care. For example, if ictal events or behaviors of interest are not captured within the standard clinical < 5-h EEG recording, the option of a longer 24 to 48-h recording could be presented to caregivers as an additional diagnostic measure (21). There is the additional possibility that dogs with more infrequent paroxysmal events might benefit from either a longer recording duration or an in-home recording if it increases the likelihood of capturing an event of interest. Further work is needed to determine the diagnostic yield of, and indications for, in-home EEG recordings.

No significant differences were observed between electrode survival time and electrode placement locations. Veterinary epileptology currently lacks a standardized electrode placement protocol (19). As no electrode location performed significantly better or worse than others, this study cannot provide any suggestions to improve upon the reported electrode placement protocol. As a next step, electrode survival time as a function of electrode location could be investigated in a larger sample size of dogs, while also optimizing source localization techniques. The impact of additional factors such as skull shape, size of dog, and coat length on electrode survival should also be considered when developing an optimal electrode placement protocol.

All actigraphy units remained secure on the EEG transmitter unit in the harness pocket on the dog's back for the duration of the recording. The most suitable accelerometer location for canine epilepsy research has yet to be determined; the two most reported and successful locations are the interscapular region or around the dog's neck (21–23, 27, 28). The current study selected the interscapular region inside the harness pocket for actigraphy placement as the unit was not easily attachable to the dog's collar and to minimize the risk of damage to the unit itself. Establishing the optimal location for actigraphy placement will require comparisons of data quality between locations to determine which placement protocol reduces the movement of the actigraphy itself, as movement or rotation of the unit may interfere with data accuracy (28). As no EEG-confirmed ictal events occurred during the recording period, we were unable to identify actigraphy values correlating to seizure activity. Non-GTC seizures may be more difficult to detect via accelerometry because they are often much less disruptive and convulsive, and the accelerometer may be unable to recognize the variable and relatively minor movements that accompany non-GTC seizures such as excessive blinking or lip licking. The inability to detect non-GTC seizures may result in the underestimation of seizure frequency, which is further compounded by the tendency for caregivers to underreport seizure incidence (7). If ictal events occur in future research, additional actigraphy outcomes such as the feasibility of algorithms to detect and classify GTC and non-GTC seizure activity could be assessed.

A wide range of questionnaire completion times was observed and ranged from 8.72 to 2,748.93 min for Q1, Q2, and Q3, with median completion times of 101.47, 16.5, and 21.94 min, respectively. The questionnaires posed in this research were completed by a small focus group (*n* = 5) of unrelated subjects prior to study initiation to help ensure they could be completed in a reasonable time. Implementing questionnaires of a reasonable length is essential to ensure the quality of the responses does not diminish over time (29). The abnormally long completion times for Q1 may have been a result of the distracting hospital environment and their dog's reaction to instrumentation. In future, this questionnaire could be completed at the caregiver's home prior to instrumentation to minimize distractions. Additional factors affecting completion time for internet-based questionnaires include participant age, experience with internet-based questionnaires, and education, which may have played a role in the longer completion times shown in this study (30). The behavioral data obtained from these questionnaires were not analyzed for the present study due to the small sample size but will serve as a foundation for a prospective study examining this technology combination in a larger sample size.

The sample size for the present study was intentionally kept small to ensure this technology combination was feasible for at-home recordings before investing significant time and monetary resources in a larger sample size. A prospective study examining the same novel technology combination would require 40 dogs to be sampled from the population to be 80% confident that the estimated proportion of feasible trials is within 10% of the true population proportion.

This feasibility study was limited by several factors. First, the geographic location limited participation as the study design required multiple visits to the OVC HSC, restricting recruitment to local caregivers or caregivers willing to make these visits irrespective of location. This study was also task heavy for caregivers with the multiple visits to the OVC HSC, competition of several questionnaires, and up to 48-h monitoring of both their dog and our equipment. It is worth noting that the demands of the study may have been off-putting for potential trial participants. A frustrating limitation involved the technology itself, resulting in the partial loss of the synchronized video data portion of the vEEGs due to issues with file size and portable computer capacity. The portion of video that was recorded was used to confirm stages of mentation against EEG output, but no additional behavioral observations were able to be made. Lastly, the present study's samples unintentionally consisted of medium and large breed dogs. Weight stipulations were not explicitly stated in the inclusion criteria, as the goal was to recruit IE cases and NT controls. Nonetheless, wireless vEEG devices will need to be smaller to accommodate smaller dog breeds and cats.

The recognition of complex behavioral comorbidities in dogs with epilepsy continues to grow in veterinary research and medicine, although the classification of these behaviors remains a challenge. Many facets of epileptic canine behavior exist, including pre-and post-ictal changes, prodromal changes in behavior, psychosocial behaviors, and behavioral manifestations of absence or focal seizures. These complex behavioral presentations support the growing need for objective behavior classification tools in canine epilepsy. Electroencephalography remains the standard for identifying and classifying seizure types and frequency due to its sufficient spatial and temporal resolution. Combined with synchronized video recording, vEEG becomes useful for behavioral observations and helps classify behavioral events as ictal or non-ictal (20). Actigraphy classifies different states of behavior such as walking, running, and sleeping, and identifies only GTC seizures in dogs (21–23, 27). Caregiver-completed questionnaires describe the dog's typical housing, care, routine, and behaviors, and provides subjective accounts of seizure semiology. Therefore, two objective tools, EEG and actigraphy, could strengthen the data obtained from the more commonly employed subjective questionnaire tool to understand epileptic canine behavior. The end goal of objective behavior classification in epileptic dogs is to improve the diagnosis and treatment of seizures and provide the caregiver with an understanding of how their dog's behavior changes relate to seizures to aid in seizure prediction.

Overall, this study shows that it is possible to record wireless ambulatory EEG in the home environment in addition to actigraphy recordings and caregiver questionnaires. These findings open the door for this combination to act as a research tool to examine behavior in canine epilepsy or as a diagnostic tool for complex presentations. Employing in-home EEG recordings and/or supplementing EEG recordings with actigraphy and the right questionnaires could provide clinicians with a more complete behavioral profile of each dog. In addition, this could aid clinicians in distinguishing behaviors as ictal or non-ictal. Ultimately, this area requires more research as understanding behavior in canine epilepsy is vital to improving clinicians' ability to diagnose and treat seizures while also aiding caregivers to predict seizures more accurately in their dogs.

## Data availability statement

The original contributions presented in the study are included in the article/Supplementary material, further inquiries can be directed to the corresponding author.

## Ethics statement

The studies involving humans were approved by Research Ethics Board at the University of Guelph. The studies were conducted in accordance with the local legislation and institutional requirements. The participants provided their written informed consent to participate in this study. The animal studies were approved by the Animal Care Committee and the Research Ethics Board at the University of Guelph. The studies were conducted in accordance with the local legislation and institutional requirements. Written informed consent was obtained from the owners for the participation of their animals in this study.

## Author contributions

EF, LN, and FJ: conception. EF, CM, LN, and LG: drafting the grant proposal. EF and CM: experimental logistics. EF and FJ: data collection and drafting the manuscript. EF, GM, and FJ: data analysis. EF, LN, LG, CM, GM, and FJ: revising the manuscript for intellectual content and final approval of the completed manuscript. All authors contributed to the article and approved the submitted version.
