# Supplementary material for: Feasibility of in-home electroencephalographic and actigraphy recordings in dogs
Source: Front Vet Sci. 2024 Jan 8;10:1240880. doi: 10.3389/fvets.2023.1240880 (PMC10800542; doi:10.3389/fvets.2023.1240880)
Supplement: Supplementary file 1 [file Data_Sheet_1.PDF]

Epilepsy and Behaviour Questionnaire-INTAKE

Start of Block: Section A: General information

General Information

Please provide your first and last name.

____

Please provide your dog's first and last name.

____

Has your dog been previously diagnosed with idiopathic epilepsy by a veterinarian?

- ○ Yes
- ○ No

End of Block: Section A: General information

Start of Block: Section B: Seizure characteristics

Seizure Characteristics

What age (in years) did your dog receive his/her idiopathic epilepsy diagnosis?

____

What do your dog's seizures look like? Please describe in the text box below, including possible triggers, changes in behaviour pre/post seizure, and changes in mentation and bodily control during the seizure.

____

____

____

____

____

On average, how long do your dog's seizures last?

- ○ Less than 10 seconds
- ○ Between 10 and 30 seconds
- ○ Between 30 seconds to 1 minute
- ○ Between 1 minute and 3 minutes
- ○ Greater than 3 minutes

Approximately how many seizures has your dog had in the last:

Hour: : _______

Day: : _______

Week: : _______

Month: : _______

Year: : _______

Total : ________

Has your dog been previously prescribed anti-seizure drugs?

○ Yes

○ No

End of Block: Section B: Seizure characteristics

Start of Block: Section B.1: Anti-seizure drugs

Seizure Treatment

What anti-seizure drugs has your dog been prescribed in the past that he/she is no longer taking? Select yes or no for each drug option.

	Yes	No
Phenobarbital	○	○
Potassium bromide	○	○
Levetiracetam	○	○
Zonisamide	○	○
Premidone	○	○
Other: please specify (write 'none' in the box below and select 'No' if not applicable)	○	○

Is your dog currently taking anti-seizure drugs?

○ Yes

○ No

End of Block: Section B.1: Anti-seizure drugs

Start of Block: Section C: Medical history

Medical History

Does your dog have any other medical conditions that have been diagnosed by a veterinarian?

○ Yes, please specify: ____

○ No

Has your dog been diagnosed with any behavioural disorders by a veterinarian or animal behaviour specialist?

○ Yes, please specify: ____

○ No

Do you have any additional concerns about your dog's health or behaviour that have not yet been identified by a veterinarian?

○ Yes, please specify: ____

○ No

End of Block: Section C: Medical history

Start of Block: Section D: Environmental preferences

Environmental Preferences

Approximately how many hours are you (or another member of your household) at home with your dog on a weekday?

- ○ Less than 4 hours
- ○ Between 4 to 8 hours
- ○ Between 8 to 12 hours
- ○ More than 12 hours

Approximately how many hours do you (or another member of your household) spend in close contact with your dog in a day (i.e., he/she is following you, sitting at your feet or on your lap, etc.)

- ○ Less than 1 hour
- ○ Between 1-3 hours
- ○ Between 3-6 hours
- ○ More than 6 hours

Approximately how many hours does your dog sleep during the day?

- ○ Less than 2 hours
- ○ Between 2-4 hours
- ○ Between 4-6 hours
- ○ Between 6-8 hours
- ○ More than 8 hours

Where does your dog sleep during the day? Select yes or no for each sleeping location.

	Yes	No
On the floor	○	○
Dog bed	○	○
Crate	○	○
Human bed	○	○
Couch/chair	○	○
Unsure	○	○
Other	○	○

Approximately how many hours does your dog sleep during the night?

- ◯ Less than 4 hours
- ◯ Between 4-8 hours
- ◯ Between 8-12 hours
- ◯ More than 12 hours

Where does your dog sleep during the night? Select yes or no for each sleeping location.

	Yes	No
On the floor	◯	◯
Dog bed	◯	◯
Crate	◯	◯
Human bed (with you)	◯	◯
Human bed (different room)	◯	◯
Couch/chair	◯	◯
Unsure	◯	◯
Other	◯	◯

Over the last 7 days, what has been your dog's ability to sleep without moving or getting up (restful sleep)? For the 6 prompts below, choose a number between 1 (never) and 10 (constant) that best describes your dog's ability to sleep.

	Never		Sometimes		About half the time		Most of the time		Constant	
	1	2	3	4	5	6	7	8	9	10
Moves (relocates to a new area in your home):										
Twitches (quick and rigid movements of the feet, legs, head, or body):										
Vocalizes:										
Dreams (twitching and vocalizing at the same time):										
Shifts position (changes sleeping position. i.e., from laying on belly to laying on side):										
Paces:										

How many meals is your dog fed in one day?

- ◯ 1 meal
- ◯ 2 meals
- ◯ 3 meals
- ◯ More than 3 meals

How does your dog typically eat their food? Select yes or no for each feeding option.

	Yes	No
Grazing	○	○
In one sitting	○	○
Puzzle feeder	○	○
Other	○	○

On average, how many times does your dog visit their water bowl in a day?

- ○ Less than 5 times
- ○ Between 5-10 times
- ○ Between 10-15 times
- ○ More than 15 times
- ○ Unsure

What kind of exercise does your dog get on a normal day? Select yes or no for each exercise option.

	Yes	No
On-leash walk	○	○
Off-leash walk	○	○
On-leash running	○	○
Off-leash running	○	○
Playtime with toys inside	○	○
Playtime with toys outside	○	○
Playtime with other dogs	○	○
Sports/agility	○	○
Other	○	○

On average, how many minutes of exercise does your dog get on a normal day?

○ Less than 30 minutes

○ 30-60 minutes

○ 60-90 minutes

○ 90-120 minutes

○ More than 120 minutes

On average, what other kinds of enrichment does your dog experience on a daily basis? Select yes or no for each enrichment activity.

	Yes	No
Chewing on bones	○	○
Food puzzles	○	○
Training	○	○
Games	○	○
Other	○	○

End of Block: Section D: Environmental preferences

Start of Block: Section E: Trainability

Trainability

Some dogs are more obedient and trainable than others. By checking the appropriate boxes, please indicate how trainable or obedient your dog has been in each of the following situations in the recent past.

	Never	Seldom	Sometimes	Usually	Always	Not observed/ not applicable
When off-leash, returns immediately when called	○	○	○	○	○	○
Obeys the "sit" command immediately	○	○	○	○	○	○
Obeys the "stay" command immediately	○	○	○	○	○	○
Seems to attend/listen closely to everything you say or do	○	○	○	○	○	○
Slow to respond to correction or reprimands, 'thick-skinned'	○	○	○	○	○	○
Slow to learn new tricks or tasks	○	○	○	○	○	○
Easily distracted by interesting sites, sounds, or smells	○	○	○	○	○	○

Will fetch or attempt to fetch sticks, balls, or objects	○	○	○	○	○	○

End of Block: Section E: Trainability

Start of Block: Section F: Aggression

Aggression

Some dogs display aggressive behaviour from time to time. Typical signs of moderate aggression in dogs include barking, growling, and baring teeth. More serious aggression generally includes snapping, lunging, biting, or attempting to bite. Please select a number on the 5-point scale to indicate your own dog's recent tendency to display aggressive behaviour in each of the following contexts.

	No aggression: no visible signs of aggression	Minimal aggression	Moderate aggression: growling, barking, baring teeth	Moderately severe aggression	Serious aggression: snaps, bites, or attempts to bite	Not observed/ not applicable
When verbally corrected or reprimanded (scolded, shouted at, etc.) by you or a household member.	○	○	○	○	○	○
When approached directly by an unfamiliar adult while being walked/exercised on a leash.	○	○	○	○	○	○
When approached directly by an unfamiliar child while being walked/exercised on a leash.	○	○	○	○	○	○
Toward unfamiliar people approaching the dog while he/she is in your car (at a gas station for example).	○	○	○	○	○	○
When toys, bones, or other objects are taken away by a household member.	○	○	○	○	○	○

When bathed or groomed by a household member.	◯	◯	◯	◯	◯	◯
When an unfamiliar person approached you or another member of your family at home.	◯	◯	◯	◯	◯	◯
When an unfamiliar person approaches you or another member of your family away from your home.	◯	◯	◯	◯	◯	◯
When approached directly by a household member while he/she (the dog) is eating.	◯	◯	◯	◯	◯	◯
When mailmen or other delivery workers approach your home.	◯	◯	◯	◯	◯	◯
When his/her food is taken away be a household member.	◯	◯	◯	◯	◯	◯
When strangers walk past your home while your dog is outside or in the yard.	◯	◯	◯	◯	◯	◯
When an unfamiliar person tries to touch or pet your dog.	◯	◯	◯	◯	◯	◯
When joggers, cyclists,	◯	◯	◯	◯	◯	◯

rollerbladers or skateboarders pass your home while your dog is outside or in the yard.						
When approached directly by an unfamiliar male dog while being walked/exercised on a leash.	○	○	○	○	○	○
When approached directly by an unfamiliar female dog while being walked/exercised on a leash.	○	○	○	○	○	○
When stared at directly by a member of the household.	○	○	○	○	○	○
Toward unfamiliar dogs visiting your home.	○	○	○	○	○	○
Towards cats, squirrels, or other small animals entering your yard.	○	○	○	○	○	○
Towards unfamiliar persons visiting your home.	○	○	○	○	○	○
When barked, growled, or lunged at by another (unfamiliar) dog.	○	○	○	○	○	○
When stepped over by a member of the	○	○	○	○	○	○

household.						
When you or a household member retrieves food or objects stolen by the dog.	○	○	○	○	○	○
Towards another (familiar) dog in your household (select 'not applicable' if no other dogs).	○	○	○	○	○	○
When approached at a favourite resting/sleeping place by another (familiar) household dog (select 'not applicable' if no other dogs).	○	○	○	○	○	○
When approached while eating by another (familiar) household dog (select 'not applicable' if no other dogs).	○	○	○	○	○	○
When approached while playing with/chewing a favourite toy, bone, object, etc., by another (familiar) household dog (select 'not applicable' if no other dogs).	○	○	○	○	○	○

Are there any other situations in which your dog is sometimes aggressive? If so, please describe briefly:

____

____

____

____

____

End of Block: Section F: Aggression

Start of Block: Section G: Fear and Anxiety

Fear and Anxiety

Dogs sometimes show signs of anxiety or fear when exposed to particular sounds, objects, persons, or situations. Typical signs of mild to moderate fear include: avoiding eye contact, avoidance of the feared object; crouching or cringing with tail lowered or tucked between the legs; whimpering or whining, freezing, and shaking or trembling. Extreme fear is characterized by exaggerated cowering, and/or vigorous attempts to escape, retreat or hide from the feared object, person or situation. Using the following 5-point scales (0=no fear, 4=extreme fear), please indicate your own dog's recent tendency to display fearful behaviour in each of the following circumstances.

	No fear/anxiety: no visible signs of fear	Minimal fear/anxiety	Mild to moderate fear/anxiety	Moderately extreme fear/anxiety	Extreme fear: cowers, retreats, hides, etc.	Not observed/ not applicable
When approached directly by an unfamiliar adult while away from your home.	○	○	○	○	○	○
When approached directly by an unfamiliar child while away from your home.	○	○	○	○	○	○
In response to sudden or loud noises (e.g. vacuum cleaner, car backfire, road drills, objects being dropped, etc.)	○	○	○	○	○	○
When unfamiliar persons visit your home.	○	○	○	○	○	○
When an unfamiliar person tries to touch or pet the dog.	○	○	○	○	○	○
In heavy traffic.	○	○	○	○	○	○

In response to strange or unfamiliar objects on or near the sidewalk (e.g. plastic trash bags, leaves, flags flapping, etc.)	○	○	○	○	○	○
When examined/treated by a veterinarian.	○	○	○	○	○	○
During thunderstorms, firework displays, or similar events.	○	○	○	○	○	○
When approached directly by an unfamiliar dog of the same or larger size.	○	○	○	○	○	○
When approached directly by an unfamiliar dog of a smaller size.	○	○	○	○	○	○
When first exposed to unfamiliar situations (e.g. first car trip, first time in elevator, first visit to veterinarian, etc.)	○	○	○	○	○	○
In response to wind or wind-blown objects.	○	○	○	○	○	○
When having nails clipped by a household member.	○	○	○	○	○	○
When groomed or bathed by a	○	○	○	○	○	○

household member.						
When having his/her feet toweled by a member of the household.	○	○	○	○	○	○
When barked, growled, or lunged at by an unfamiliar dog.	○	○	○	○	○	○

End of Block: Section G: Fear and Anxiety

Start of Block: Section H: Separation-related behaviour

Separation-Related Behaviour

Some dogs show signs of anxiety or abnormal behaviour when left alone, even for relatively short periods of time. Thinking back over the recent past, how often has your dog shown each of the following signs of separation-related behaviour when left, or about to be left, on its own (check appropriate boxes).

	Never	Seldom	Sometimes	Usually	Always	Not observed/ not applicable
Shaking, shivering, or trembling	○	○	○	○	○	○
Excessive salivation	○	○	○	○	○	○
Restlessness/agitation/pacing	○	○	○	○	○	○
Whining	○	○	○	○	○	○
Barking	○	○	○	○	○	○
Howling	○	○	○	○	○	○
Chewing/scratching at doors, floor, windows, curtains, etc.	○	○	○	○	○	○
Loss of appetite	○	○	○	○	○	○

Are there any other situations in which your dog is fearful or anxious? If so, please describe:

____

____

____

____

____

End of Block: Section H: Separation-related behaviour

Start of Block: Section I: Excitability

Excitability

Some dogs show relatively little reaction to sudden or potentially exciting events and disturbances in their environment, while others become highly excited at the slightest novelty. Signs of mild to moderate excitability include increased alertness, movement toward the source of novelty, and brief episodes of barking. Extreme excitability is characterized by a general tendency to over-react. The excitable dog barks or yelps hysterically at the slightest disturbance, rushes towards and around any source of excitement, and is difficult to calm down. Using the following 5-point scales please indicate your own dog's recent tendency to become excitable in each of the following circumstances.

	Calm: little or no special reaction	Minimal excitability	Mild to moderate excitability	Moderately extreme excitability	Extremely excitable: over-reacts, hard to calm down	Not observed/not applicable
When you or other members of the household come home after a brief absence.	○	○	○	○	○	○
When playing with you or other members of your household.	○	○	○	○	○	○
When doorbell rings.	○	○	○	○	○	○
Just before being taken for a walk.	○	○	○	○	○	○
Just before being taken on a car trip.	○	○	○	○	○	○
When visitors arrive at your home.	○	○	○	○	○	○

Are there any other situations in which your dog sometimes becomes over-excited? If so, please briefly describe:

____

____

____

____

____

End of Block: Section I: Excitability

Start of Block: Section J: Attachment and Attention-Seeking

Attachment and Attention-Seeking Behaviour

Most dogs are strongly attached to their people, and some demand a great deal of attention and affection from them. Thinking back over the recent past, how often has your dog shown each of the following signs of attachment or attention-seeking.

	Never	Seldom	Sometimes	Usually	Always	Not observed/not applicable
Displays a strong attachment for one particular member of the household.	○	○	○	○	○	○
Tends to follow you (or other members of the household) about the house, from room to room.	○	○	○	○	○	○
Tends to sit close to, or in contact with, you (or others) when you are sitting down.	○	○	○	○	○	○
Tends to nudge, nuzzle, or paw you (or others) for attention when you are sitting down.	○	○	○	○	○	○
Becomes agitated (whines, jumps up,	○	○	○	○	○	○

tries to intervene) when you (or others) show affection for another person.						
Becomes agitated (whines, jumps up, tries to intervene) when you show affection for another dog or animal.	○	○	○	○	○	○

End of Block: Section J: Attachment and Attention-Seeking

Start of Block: Section K: Miscellaneous

Miscellaneous Behaviours

Dogs display a wide range of miscellaneous behaviour problems in addition to those already covered by this questionnaire. Thinking back over the recent past, please indicate how often your dog has shown any of the following behaviours:

	Never	Seldom	Sometimes	Usually	Always	Not observed/ not applicable
Chases or would chase cats given the opportunity.	○	○	○	○	○	○
Chases or would chase birds given the opportunity.	○	○	○	○	○	○
Chases or would chase squirrels, rabbits, or other small animals given the opportunity.	○	○	○	○	○	○
Escapes or would escape from home or yard given the chance.	○	○	○	○	○	○
Rolls in animal droppings or other 'smelly' substances.	○	○	○	○	○	○
Chews inappropriate objects.	○	○	○	○	○	○
'Mounts' objects, furniture, or people.	○	○	○	○	○	○
Begs persistently for food when people are eating.	○	○	○	○	○	○
Steals food.	○	○	○	○	○	○
Nervous or frightened on stairs.	○	○	○	○	○	○

Pulls excessively hard when on the leash.	○	○	○	○	○	○
Urinates against objects/furnishings in your home	○	○	○	○	○	○
Urinates when approached, handled, or picked up.	○	○	○	○	○	○
Urinates when left alone at night or during the daytime.	○	○	○	○	○	○
Hyperactive, restless, has trouble settling down.	○	○	○	○	○	○
Playful, puppyish, boisterous.	○	○	○	○	○	○
Active, energetic, always on the go.	○	○	○	○	○	○
Stares intently at nothing visible.	○	○	○	○	○	○
Snaps at (invisible) flies.	○	○	○	○	○	○
Chases own tail/ hind end.	○	○	○	○	○	○
Chases/ follows shadows, light spots, etc.	○	○	○	○	○	○
Barks persistently when alarmed or excited.	○	○	○	○	○	○
Licks him/herself excessively.	○	○	○	○	○	○
Licks people or objects excessively.	○	○	○	○	○	○

Displays other bizarre, strange, or repetitive behaviour (s). Please describe in the box below. If there are no other strange behaviours, please write none in the box below and select the 'not applicable' option.	○	○	○	○	○	○

End of Block: Section K: Miscellaneous

Start of Block: Additional remarks

Please use this space to provide any additional information about your dogs habits, behaviour, and environmental preferences. Please also use this space to provide feedback regarding the questionnaire experience.

____

____

____

____

____

Proceeding forward will submit your questionnaire responses. Please ensure you have answered all questions to your liking before proceeding.

End of Block: Additional remarks

Start of Block: Section C.1: History of medications

History of Medications

Is your dog currently taking any medications, supplements, or other prescribed treatments for these conditions?

○ Yes: please specify ____

○ No

End of Block: Section C.1: History of medications

Start of Block: Section B.2: Current anti-seizure treatments

Current Seizure Treatments

What anti-seizure drugs is your dog currently taking? Select all that apply.

▢ Phenobarbital

▢ Potassium bromide

▢ Levetiracetam

▢ Zonisamide

▢ Premidone

▢ Other: please specify ____

End of Block: Section B.2: Current anti-seizure treatments
