# Supplementary material for: Feasibility of in-home electroencephalographic and actigraphy recordings in dogs
Source: Front Vet Sci. 2024 Jan 8;10:1240880. doi: 10.3389/fvets.2023.1240880 (PMC10800542; doi:10.3389/fvets.2023.1240880)
Supplement: Supplementary file 4 [file Table_2.DOCX]

| ID | Breed | IE or NT | Sex | Age (years) | Weight (kg) | EEG recording duration (hours) | Actigraphy recording duration (hours) |
| --- | --- | --- | --- | --- | --- | --- | --- |
| DOG1 | Border Collie | NT | F | 2 | 12.9 | 46.8 | 47.0 |
| DOG2 | Standard Poodle | IE | F | 4 | 23.9 | 46.9 | 46.8 |
| DOG3 | Golden Retriever | IE | F | 5 | 41 | 27.7 | 25.6 |
| DOG4 | Labrador Retriever Mix | IE | M | 5 | 33.2 | 48.3 | 48.0 |
| DOG5 | Golden Retriever Mix | IE | M | 3 | 32.9 | 44.1 | 44.95 |
| DOG6 | Labrador Retriever Mix | NT | M | 7 | 33.4 | 17.4 | 6.7 |
| DOG7 | Black Mouth Cur | IE | M | 7 | 28.5 | 45.3 | 21.5 |
| DOG8 | Labrador Retriever Mix | NT | F | 3 | 31.6 | 1.9 | 1.7 |
| DOG9 | Great Pyrenees Mix | IE | M | 5 | 38.6 | 2.3 | 8.9 |
| DOG10 | Husky Mix | NT | F | 8 | 28.5 | 2.8 | 4.2 |

Supplementary Table 2.

A detailed description of the participating dog’s signalment including breed, group (IE or NT), sex, age, weight, and EEG and actigraphy recording durations.
